# Topical Capsaicin for Treating Cannabinoid Hyperemesis Syndrome

**DOI:** 10.1155/2020/8868385

**Published:** 2020-11-27

**Authors:** Ansar Aziz, Tayyab Waheed, Olubunmi Oladunjoye, Adeolu Oladunjoye, Midhat Hanif, Fareena Latif

**Affiliations:** ^1^Reading Hospital Tower Health, West Reading, PA, USA; ^2^Boston Children's Hospital, Boston, MA, USA; ^3^Independent Researcher, West Reading, PA, USA

## Abstract

**Introduction:**

Cannabinoid hyperemesis syndrome (CHS), associated with chronic cannabis use, presents with cyclic abdominal pain, nausea, and vomiting. With increasing use of marijuana, the incidence of CHS is expected to increase. Most patients with CHS make frequent visits to the emergency room and are usually refractory to conventional treatment. We, therefore, present a case of CHS successfully treated with topical capsaicin application. *Case Presentation*. A 41-year-old female with a recent excess use of cannabis presented to the emergency department for evaluation of recurrent excruciating epigastric pain accompanied by severe nausea and vomiting. She had similar, milder symptoms a year ago and underwent endoscopic evaluation which was negative except for mild reflux esophagitis for which she was started on a proton pump inhibitor. On this presentation, basic laboratory workup, EKG, and CT scan of abdomen and pelvis were unremarkable. A detailed abdominal exam was only positive for mild epigastric tenderness. She was instructed to continue pantoprazole and pain medication and outpatient repeat esophagogastroduodenoscopy. The patient returned the next day with extreme retching, nausea, and vomiting and was admitted for further evaluation. Intravenous fluids, antiemetics, and morphine were started for pain control with no symptomatic improvement. A diagnosis of cannabis hyperemesis syndrome was made based on history of chronic marijuana use and otherwise negative workup. A trial of topical capsaicin, over the epigastric region, was tried that provided dramatic relief within 24 hours. Repeat endoscopic evaluation had no evidence of ulcers, celiac disease, or esophagitis. She was discharged on topical capsaicin and counselled on marijuana abstinence, with no return of symptoms.

**Conclusion:**

Based on the dramatic resolution of symptoms with topical capsaicin, our case supports this promising intervention and provides an alternate approach to antiemetics and narcotics routinely used in patients with cannabis hyperemesis syndrome.

## 1. Introduction

Cannabinoid hyperemesis syndrome (CHS) is a known condition associated with chronic cannabis use. It usually presents with cyclic abdominal pain, nausea, and vomiting and often followed by a temporary relief with a hot shower bath. As increased use of marijuana is observed since its legalization, the incidence of CHS is expected to be on the rise [1–4]. Unfortunately, most patients with CHS make frequent visits to the emergency room and get several medical tests and imaging studies done which eventually turn out to be negative or lead to delay in diagnosing CHS [5–8]. In addition, some of these patients end up receiving multiple medications including antiemetics with potential side effects and are still refractory to treatment [6, 9].

The pathophysiology of CHS is not well understood. However, there are receptors implicated called transient receptor potential vanilloid 1 (TRPV-1) receptors which respond to high temperature and substance P regulation (a nausea and vomiting mediator). This might explain the reason why hot shower bath provides temporary symptomatic relief in CHS patients [5, 10, 11]. TRPV-1 is also found in the peripheral nervous system and is thought to be involved in dysregulation of the endocannabinoid system in the hypothalamus. Capsaicin is known to exert its effects on this system and can potentially provide symptomatic relief [12, 13].

Here, we present a case of CHS successfully treated with topical capsaicin application.

## 2. Case Report

A 41-year-old female with significant past medical history of cannabis use, tobacco use, bipolar disorder, chronic obstructive pulmonary disease, hypertension, and reflux esophagitis, but no alcohol use, presented to the emergency department for evaluation of recurrent excruciating epigastric pain accompanied by severe nausea and vomiting. Previously, she has had a similar but milder symptom for which she was evaluated. Previous esophagogastroduodenoscopy revealed no evidence of Barrett's esophagus or sprue; however, reflux esophagitis was detected for which she was started on a proton pump inhibitor.

The patient had been relatively symptom-free over the course of the two years until recently when she started experiencing severe epigastric pain with retching and nausea. She was then given pantoprazole and was discharged to follow up as an outpatient. The patient returned to the emergency department a few days later with similar epigastric pain with nausea and vomiting. Electrocardiogram (EKG) was without any significant ST or T wave changes ruling out acute coronary artery syndrome. CT scan abdomen and pelvis was performed and was negative for any acute pathology. The patient was treated with pantoprazole and pain medications and was later discharged after stabilization. The next day, the patient returned to the emergency department complaining of severe crampy abdominal pain with severe nausea and intermittent vomiting. The patient described her pain as being more severe with a rating of 10/10, twisting in nature, and some sensations of “butterflies” going up in the chest. Her pain was worse with eating, and she had been unable to tolerate any solid or liquid diet. She reported no relief with pantoprazole. Her review of systems was negative for heart burn or reflux-type symptoms, diarrhea, melena, or hematochezia. She also denied headache or history of migraine.

A detailed abdominal examination was also done which was negative for guarding or rebound tenderness. The patient, however, had moderate tenderness to palpation in her epigastrium. Other workups including EKG, complete blood count (CBC), electrolytes, urinalysis, and kidney and liver functions were all within normal limits. The urinary pregnancy test was also negative.

The patient was then admitted to the hospital with initial differential diagnosis of gastritis, severe gastroesophageal reflux, cyclic vomiting syndrome, and peptic ulcer disease. She was given intravenous fluids, antiemetics, and initial one dose of morphine to decrease the pain and anxiety. The next morning, the patient's symptoms were unchanged. A urine drug screen was ordered which came back positive for marijuana. The patient admitted to its chronic use with increasing use over the past 2 weeks.

Concerns for cannabis hyperemesis syndrome were raised given the repeated bouts of abdominal pain, nausea, and vomiting with benign abdominal exam in the setting of recent increased use of marijuana. The patient was continued on pantoprazole with addition of metoclopramide for nausea control but there was no improvement in her symptoms. Esophagogastroduodenoscopy was repeated that was evident only for mild gastropathy that could not fully explain her symptoms. After discussion with the patient and her spouse, a trial of capsaicin 0.1% topical cream, one application 3 times daily, was ordered with which the patient had dramatic relief with near complete resolution of her symptoms.

## 3. Discussion

CHS is subdivided into three phases: prodromal, vomiting, and recovery phases. The prodromal phase is characterized by nausea and abdominal discomfort. The vomiting phase is characterized by severe nausea, vomiting, and retching that can occur several times in an hour [14]. During the vomiting phase, the patient shows compulsive desire to bath in hot water which often times gives them temporary relief. However, most patients will present in the emergency room when they no longer can control their symptoms [15]. The recovery phase is when the patient stops taking cannabis and begins to see cessation of symptoms within a week [14, 16, 17].

The pathophysiology of CHS is not well understood. However, in the available literature, it has been proposed that CHS maybe caused by dysregulation of endocannabinoid systems called endogenous cannabinoid receptors CB-1 and CB-2 found in the brain, peripheral nervous system, and gastrointestinal system [16]. These receptors such as CB-1 receptors are found close to TRPV-1 which might explain their effective relationship [18]. The accumulation of tetrahydrocannabinol (THC), the active component of marijuana, is thought to bind to the receptors in the gastrointestinal tract leading to gastrointestinal motility, nausea, and vomiting, hence the presentation in CHS [5, 12, 19–21].

TRPV-1 is also found in the peripheral nervous system and is thought to be involved in the dysregulation of the endocannabinoid system in the hypothalamus, and capsaicin is known to exert its effects on this system [12, 13]. Capsaicin binds TRPV-1 with high specificity and responds to high temperature and substance P regulation [22]. Capsaicin is thought to inhibit substance P action at the postrema area by overactivation of TPRV-1 and leading to antiemesis [4].

Recent trials have emerged with the use of capsaicin or 8 methyl-*N*-vanillyl-6- nonenamide, an active ingredient of chili pepper as a viable option to treat CHS [4]. It is an alkaloid extract from capsicum a flowering plant [10]. It is available in different concentrations such as 0.025 0.075, and 0.1% topical cream, and it is being reported that higher concentration formulations are more effective than lower concentration formulations [22, 23]. Capsaicin is used as a topical cream that produces heat sensation on the skin, thereby relieving significant symptoms of nausea and vomiting in CHS patients. An advantage of capsaicin is that it is noninvasive and cost-effective and has a low risk of adverse effects. However, capsaicin is not without side effects. Such side effects include burning sensation, skin irritation, and blisters at application site. Others less common side effects are diarrhea, nausea, back pain, dizziness, headache, and hypertension [24].

In a case series in 2016 by Burillo-Putze and colleagues [25, 26], which supports our study findings, they reported successful resolution of symptoms after other antiemetics such as metoclopramide and granisetron failed. Another study in 2017 by Dezieck et al. [4] reported similar findings that after the use of several antiemetics failed, their patients experienced resolution of symptoms with the use of topical capsaicin. In the case of our patient, she received antiemetics earlier with no relief which warranted the physicians to change to topical capsaicin that resulted in near resolution of symptoms. Some case reports and case series have also suggested the use of haloperidol to be effective [27–30].

A documented limitation of capsaicin use is the patient's insight and compliance to capsaicin application. It is reported that some patients could not relate their relief of symptoms to the use of topical capsaicin [4]. This may be attributed to their impaired judgement because of their chronic cannabis use. Another potential limitation is that topical capsaicin may also have affected pain sensitivity by acting on TRPV-1 receptors. However, the main stay of treatment remains cessation of cannabis use. Future studies and trials are needed to further explore the role of capsaicin use in cannabinoid hyperemesis syndrome.

## 4. Conclusion

Use of topical capsaicin for treatment of CHS seems promising as previously reported in several case reports and studies. However, further studies are warranted to support this finding to be used as a first-line treatment option for CHS management.
